# A non-circadian role for clock-genes in sleep homeostasis:a strain comparison

**DOI:** 10.1186/1471-2202-8-87

**Published:** 2007-10-18

**Authors:** Paul Franken, Ryan Thomason, H Craig Heller, Bruce F O'Hara

**Affiliations:** 1 Department of Biological Sciences, Stanford University, Stanford, CA, USA; 2 Department of Biology, University of Kentucky, Lexington, KY, USA; 3 Center for Integrative Genomics, University of Lausanne, Lausanne, Switzerland

## Abstract

**Background:**

We have previously reported that the expression of circadian clock-genes increases in the cerebral cortex after sleep deprivation (SD) and that the sleep rebound following SD is attenuated in mice deficient for one or more clock-genes. We hypothesized that besides generating circadian rhythms, clock-genes also play a role in the homeostatic regulation of sleep. Here we follow the time course of the forebrain changes in the expression of the clock-genes *period *(*per*)-*1, per2*, and of the clock-controlled gene *albumin D-binding protein *(*dbp*) during a 6 h SD and subsequent recovery sleep in three inbred strains of mice for which the homeostatic sleep rebound following SD differs. We reasoned that if clock genes are functionally implicated in sleep homeostasis then the SD-induced changes in gene expression should vary according to the genotypic differences in the sleep rebound.

**Results:**

In all three strains *per *expression was increased when animals were kept awake but the rate of increase during the SD as well as the relative increase in *per *after 6 h SD were highest in the strain for which the sleep rebound was smallest; i.e., DBA/2J (D2). Moreover, whereas in the other two strains *per1 *and *per2 *reverted to control levels with recovery sleep, *per2 *expression specifically, remained elevated in D2 mice. *dbp *expression increased during the light period both during baseline and during SD although levels were reduced during the latter condition compared to baseline. In contrast to *per2*, *dbp *expression reverted to control levels with recovery sleep in D2 only, whereas in the two other strains expression remained decreased.

**Conclusion:**

These findings support and extend our previous findings that clock genes in the forebrain are implicated in the homeostatic regulation of sleep and suggest that sustained, high levels of *per2 *expression may negatively impact recovery sleep.

## Background

At the cellular level, circadian rhythms are thought to be generated by transcriptional-translational feedback loops made up of positively and negatively acting transcriptional regulators [1]. In mammals, the core positive elements are CLOCK and NPAS2 and their obligate dimerization partner BMAL1. These transcription factors drive *per *and *cry *transcription. PER and CRY proteins, in turn, interact with CLOCK/NPAS2:BMAL1 heterodimers to inhibit their own transcription, thus constituting the negative feedback elements. The circuitry is clearly more complex as additional feedback loops and post-translational modifications are involved [1]. Clock genes underlie circadian rhythm generation in many tissues, but the circadian rhythm in the suprachiasmatic nucleus (SCN) is required for the manifestation of overt physiological and behavioral rhythms, and is therefore considered the master circadian pacemaker [1,2].

Although the role of clock genes in generating circadian rhythms is firmly established, we and others have found that they also play a role in the homeostatic regulation of non-REM sleep (NREMS) [3-5]. This notion is based on the observation that mice deficient for one or more clock genes show an altered response to sleep deprivation (SD) and on the observation that clock-gene expression in brain areas outside the SCN, notable the cerebral cortex, is influenced by the prior occurrence of sleep and wake. For instance, the typical responses in NREMS to SD were reduced or absent in mice lacking the *cry1 *and *-2 *(*cry1,2*^-/-^) [4] or *bmal1 *(*bmal1*^-/-^) [6] genes compared to wild-type controls. The diminished NREMS variables included duration, consolidation, and EEG delta power. EEG delta power quantifies the prevalence and amplitude of delta oscillations and is widely used to index the homeostatic need for NREMS [7]. Sleep under undisturbed, baseline conditions was also altered in mutant mice. *clock*-mutant (*clock*^*Δ/Δ*^) [8] and *npas2*^-/-^[3] mice spent less time in NREMS whereas *cry1,2*^-/-^[4] and *bmal1*^-/- ^[6] mice slept more. NREMS consolidation was increased in *cry1,2*^-/- ^mice while the opposite was observed in *clock*^*Δ/Δ *^[8] and *bmal1*^-/- ^[6] mice and in mice lacking *albumin D-binding protein *(*dbp*^-/-^)[9]; a gene under the transcriptional control of CLOCK [10] and that also has been implicated in circadian rhythm generation [11]. Average levels of EEG delta power were reduced in *clock*^*Δ/Δ *^[8], *dbp*^-/-^[9], and *bmal1*^-/- ^[6] mice and increased in *cry1,2*^-/- ^[4] mice.

Clock-gene expression in the brain, notably the cerebral cortex, depends on the sleep-wake (or rest-activity) distribution. Cortical levels of *per *are high at times when sleep drive is high, irrespective of the phase at which the circadian expression of *per *peaks in the SCN. Thus, in both nocturnal and diurnal species, *per *expression in the cortex is maximal in conjunction with the major waking episode [12-14]. Under conditions where the phase (methamphetamine administration, restricted feeding) or distribution (circadian splitting) of locomotor activity is altered, *per *expression in the cortex parallels the overt rhythm of wakefulness, while *per *expression in the SCN remains unaffected [13,15-17]. Consistent with these observations, our data demonstrate that *per *expression increases when mice are kept awake [3,4]. The lack or alteration of particular clock genes in the various mouse models mentioned above, changes the expression levels of other clock genes in the circuitry. For instance, basal *per *expression levels are decreased in *clock*^*Δ/Δ *^mice [10,18] and increased in *cry1,2*^-/- ^mice [4,19-21] which corresponds to their opposing sleep phenotypes (see above). Also, the SD-induced increase in *per *expression is reduced in *npas2*^-/- ^mice [3] which suggest that NPAS2 is implicated in linking *per2 *expression to the sleep-wake distribution. Likewise, *npas2*^-/- ^mice lack circadian rhythms in cortical *per2 *expression despite an intact circadian organization of sleep and waking [12].

The finding that NPAS2 couples wakefulness to clock gene expression is of particular interest given the evidence that NPAS2 (and CLOCK) -mediated transcription is redox sensitive [22]. Redox sensitivity suggests that this clock gene network may be linked to cellular energy metabolism [23]. Since restoration of an optimal neural energy state has long been considered a possible function of sleep [24], this gene network might underlie a fundamental aspect of sleep homeostasis. If so, another critical gene in this network is likely to be *ldha *(*lactate dehydrogenase A*). *ldha *can be induced in cell culture by NPAS2, has multiple E-box sites, is a critical enzyme for neuronal energy metabolism, and utilizes NADH as a co-factor [12,22]. Therefore, we decided to investigate gene expression in the forebrain in greater detail over the course of a SD and after recovery sleep for the CLOCK and NPAS2 target genes *per1*,*per2*, *dbp*, and *ldha*. Futhermore, we undertook this examination of the sleep-wake dependent changes in gene expression in three inbred strains of mice known to differ in the rate at which sleep need, quantified as EEG delta power, increases during wakefulness [25]. We reasoned that if clock genes are implicated in sleep homeostasis, then the time course of changes in gene expression during a SD in these strains should vary according to the published differences in EEG delta power dynamics.

## Results

### Control conditions

**Figure 1:**
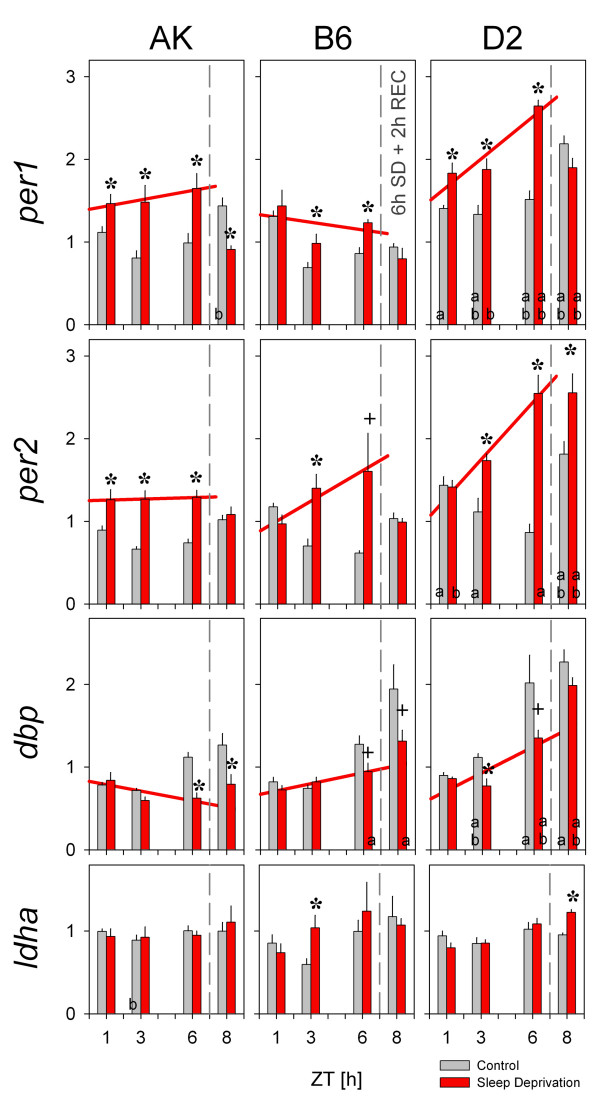
Mean (+1SEM) forebrain mRNA levels of *per1*, *per2*, *dbp*, and *ldha *(top to bottom panels) for AK (left), B6 (middle) and D2 (right panels) mice after sleep deprivations (SD) of 1-, 3-, and 6 h (red bars). All SDs started at light-onset (ZT0). Control animals (grey) were sacrificed along with the SD animals. Effects of recovery sleep on expression were assessed by allowing 2 h of recovery (REC) after 6 h SD (ZT8). *per1*, *per2*, and *dbp*, but not *ldha *expression, was affected by SD, genotype, and time-of-day (3-way ANOVA factors 'SD' and 'genotype': *P *< 0.0001, factor 'ZT': *per1 P *= 0.0002, *per2 P *= 0.004, *dbp P *< 0.0001). For both *per *genes the SD effect differed among genotypes ('SD'-'genotype' interaction: *per1*: *P *= 0.039; *per2*: *P *= 0.006). Linear regression lines (red) describe the time course of mRNA changes during SD (ZT1 through ZT6; see Fig.2). Asterisks and crosses mark times when SD differed from controls (*P *< 0.05 and *P *< 0.10, respectively; post-hoc *t*-tests). Symbols 'a' (> AK) and 'b' (> B6) mark strain differences for each time point and condition (*P *< 0.05; post-hoc Tukey).

Under undisturbed control conditions, forebrain levels of *per1*, *per2*, and *dbp *expression varied both as a function of time-of-day and of genotype (2-way ANOVA factors 'genotype' and 'ZT' *P *< 0.0001). Generally, all three genes reached highest expression levels at ZT8 (post-hoc Tukey; *P *< 0.05; Fig.1), but there were time-course differences. The values for *dbp *expression were lowest at ZT1 and ZT3 and then increased throughout the light period. In contrast, the expression of *per1 *and *per2 *first decreased from ZT1 reaching lowest levels at ZT3 or -6 before increasing. The time courses observed here match well those previously published for most brain areas outside the SCN and for certain other peripheral tissues [16,26-28] with lowest mRNA values observed early (*dbp*) or mid (subjective) day (*per1*, *per2*) and peak levels being reached around ZT8 for *dbp *or around ZT12-14 for *per1 *and *per2 *(not assessed here). No systematic variation in *ldha *expression levels was observed across the 4 time points in any strain.

**Figure 2:**
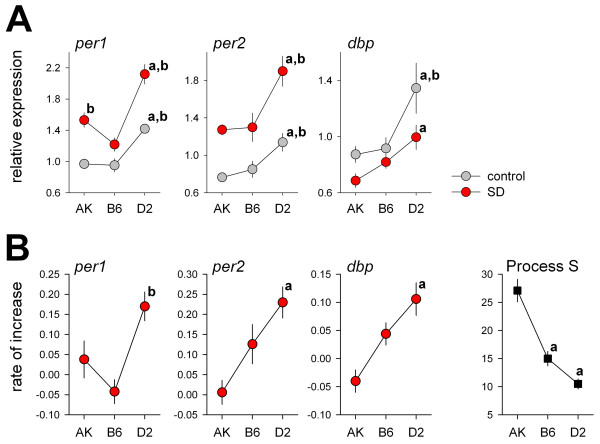
**A**) Average mRNA levels under both control (mean ZT1-6; ± 1SEM; n = 12/strain; grey) and SD (mean ZT1-6; red symbols; n = 12/strain) conditions were generally higher in D2 mice compared to AK and B6 mice ['a' (> AK), 'b' (> B6) *P *< 0.05; post-hoc Tukey]. No genotype differences were observed for *ldha *expression (not shown). **B**) Mean (± 1SEM; n = 12/genotype) slope of the regression lines quantifying the relationship between SD duration (1-, 3-, and 6 h) and clock-gene expression (fraction/h; see Fig.1) in the forebrain of AK, B6, and D2 mice. Changes in expression were affected by genotype (2-way ANOVA: 'genotype'-'ZT' interaction: *per1*: *P *= 0.0002; *per2*: *P *= 0.032; *dbp*: *P *= 0.0002). Slopes were steepest for D2 mice and the strain distribution pattern for this phenotype seemed to negatively correlate with the increase of EEG delta power (%/h) during wakefulness (i.e., 'Process S' in lower right panel; modified from [25]; with permission).

Although the time-dependent changes in gene expression did not differ among the 3 inbred strains, genetic background did affect overall expression levels within the control group. With few exceptions, expression of *per1*, *per2*, and *dbp *was higher in D2 compared to AK and B6 mice at all four time-points (post-hoc Tukey; *P *< 0.05; Fig.1, summarized in Fig.2A). Elevated expression levels for D2 were maintained even during SD-conditions (Fig.2A). Genotype did not affect the level of *ldha *expression.

### Sleep deprivation

Against this varying background the effects of enforced wakefulness on the expression of clock and clock-controlled genes was evaluated. Confirming our previous results in B6 mice [3,4], 6 h SD increased *per1 *and *per2 *expression and decreased *dbp *expression in comparison to time controls (Fig.1). The present experiment allowed us to follow the time course of gene expression during the 6 h SD and also enabled us to assess and compare the dynamics among mice of different genetic backgrounds.

SD changed the expression of these genes in the same direction in all three strains, however, significant genotype differences were observed in the dynamics and the magnitude of these changes (Figs.1 and 2B). In AK mice *per1 *and *per2 *expression was significantly increased after only 1 h of SD compared to control levels at ZT1. In D2 mice only *per1 *was increased after 1 h of SD, and in B6 mice neither *per1 *nor *per2 *were increased by 1 h of SD. However, *per1 *and *per2 *mRNA in AK mice did not increase further as SD progressed whereas in D2 mice (as well as in B6 for *per2*) a progressive increase was observed from 1- to 3- to 6 h SD (Figs.1 and 2B). Forebrain mRNA levels of *dbp *increased over the 6 h of the experiment in both control and SD groups of D2 and B6 mice, but the SD values tended to be lower than the control values after 3 and 6 h SD. The increase of *per1*, *per2*, and *dbp *over the course of the SD was quantified by linear regression analysis which demonstrated that the rate of increase was significantly greater in D2 mice compared to AK mice (Figs.1 and 2B). The largest changes (both increases and decreases) in mRNA levels were observed after 6 h SD. For *per1 *and *per2 *expression the increase at this time point differed significantly among inbred strains (2-way ANOVA interaction 'genotype'-'SD': *per1 P *= 0.009; *per2 P *= 0.022). In D2 mice the *per1 *increase (1.8-fold, ± 0.3) was larger than in B6 mice (1.4 ± 0.1; *P *= 0.0003) and the *per2 *increase (2.9 ± 0.3) larger than in AK mice (1.8 ± 0.1; *P *= 0.0010). Sleep deprivation did not affect *ldha *expression.

The genotype specific changes in clock-gene expression during SD (Fig.2B), the genotype differences in the average expression levels during both control and SD (Fig.2A), and the larger relative increase in *per1 *and *per2 *after 6 h SD are all opposite to the strain differences we previously reported in the dynamics of the homeostatic regulation of sleep [25]. For this phenotype the rate of increase in sleep need during wakefulness (quantified as EEG delta power during NREMS) was fastest in AK mice and slowest in D2 mice (Fig.2B). This suggests a negative correlation between the magnitude of the increase in these clock genes and the homeostatic regulation of sleep. To facilitate a direct comparison between our present and our previous study we made every effort to follow the same experimental protocol and conditions including the LD cycle, light intensity, ambient temperature, animal provider, sex and age of the mice, acclimatization time, start time and method of sleep deprivation.

### Recovery sleep

Two hours of recovery sleep affected *per1 *and *per2 *forebrain mRNA levels in a strain specific manner (Fig.1). *per1 *expression sharply decreased from the levels reached at the end of the 6 h SD and in AK mice values reached at ZT8 even fell below control levels. A similar recovery pattern was observed for *per2 *expression with the exception of D2; in this strain, recovery sleep could not lower *per2 *mRNA levels and values remained significantly elevated compared to control. Conversely, only in D2 mice did the decrease in *dbp *expression revert to control levels.

### Correlations

**Figure 3:**
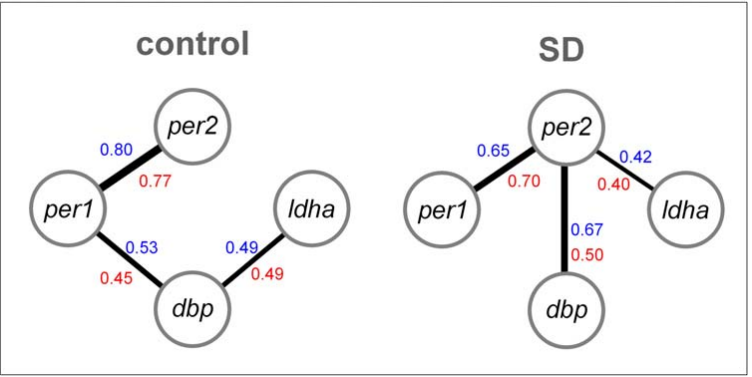
Significant correlations among forebrain expression levels of the four genes examined during control (left; n = 48) and sleep deprivation (SD; right; n = 48) conditions. Only those correlations between gene pairs are indicated that remained significant after partialling out the influence of the other two. In blue the Pearson's correlation coefficients, in red the partial correlation coefficients. Line thickness corresponds to correlation strength.

The expression of all four genes studied here are regulated by CLOCK and NPAS2 through E-boxes [22,29-31] although other cis-acting elements are known to be involved; e.g., cAMP-response and DBP-binding elements [11,26,29,32,33]. Given the likelihood of shared transcriptional regulation through E-boxes, we tested expression patterns for similarities among these four genes and how these relationships were affected by the SD (Fig.3). Under both control and SD conditions the two *period *genes were strongly correlated sharing 63 and 42% of the variance, respectively (R^2^; linear regression, *P *< 0.0001; Fig.3). Other significant correlations observed during control conditions were between *per1 *and *dbp *(R^2 ^= 0.28; *P *< 0.0001) and between *dbp *and *ldha *(R^2 ^= 0.24; *P *= 0.0004) expression. These relationships changed dramatically with SD; *per2 *expression, which was affected the most by SD, now became correlated with that of the other 3 genes while the *per1*-*dbp *and *dbp*-*ldha *correlations no longer reached significance levels and the *per2*-*dbp *correlation, that was not significant during control, became equally important as the *per1*-*per2 *correlation (R^2 ^= 0.44 and 0.42 respectively, *P *< 0.0001; Fig.3). The SD-induced increase in the correlation coefficient between *per2 *and *dbp *expression (-0.15 vs. 0.50) and the decrease observed for the *per1*-*dbp *correlation (0.45 vs. -0.12) were significant (*P *< 0.005; 2-sided; n = 48/condition). Within strains, the same relationships were observed, although they did not always reach significance levels (analyses not shown).

### In situ hybridization

**Figure 4:**
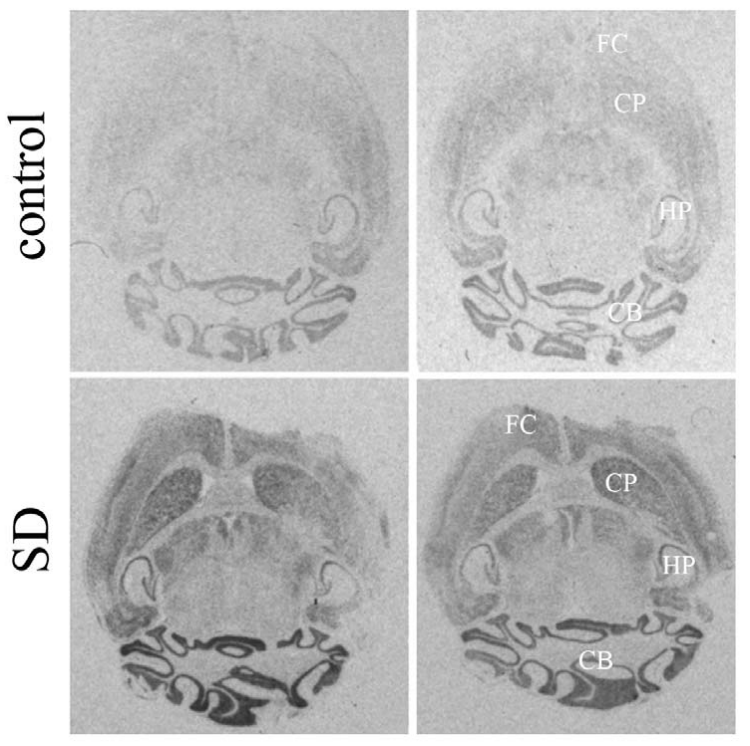
Representative *in situ *hybridizations for *per1 *expression in two horizontal brain sections from a sleep deprived (SD, lower panels) and a control (upper panels) mouse. Following 6 h SD *per1 *mRNA increased throughout the brain. This increase was especially pronounced in the cerebral cortex and cerebellum (CB), consistent with our qPCR data (Fig. 1). Note the very low cortical expression in control mice which, at ZT6, spent most of the preceding 6 h sleeping. Within the cortex, increases were especially notable in the frontal cortex (FC) and in and around the cingulate cortex. Caudate-Putamen (CP) and hippocampus (HP) are labeled for orientation.

**Figure 5:**
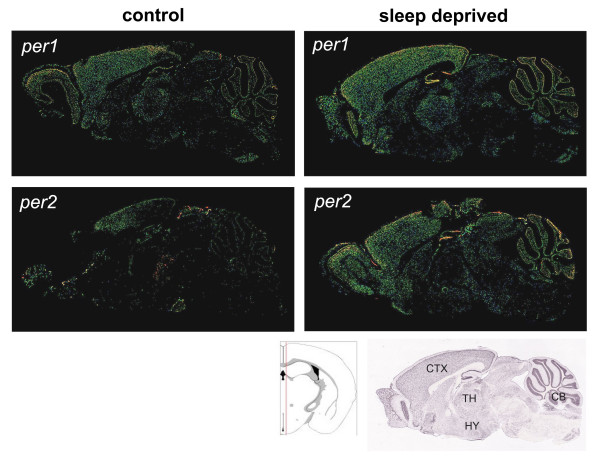
*In situ *hybridizations for *per1 *(upper) and *per2 *(middle panels) expression in four sagittal brain sections from sleep deprived (SD, right) and control (left panels) mice. All sections were taken close to mid-line (see lower panel; red vertical line in coronal section indicates the plane of the sagittal sections). *per *was expressed mostly in cortex and cerebellum and SD induced an increase in both these areas, confirming our own *per1 *expression data (Fig. 4). These data were taken, with permission, from the publicly available *in situ *studies of the Allen Institute for Brain Science [60], done in collaboration with Drs. T.S. Kilduff and J.P. Wisor of SRI International. The experimental protocol was identical to that of the 6 h SD in the present study; both SD and control animals were sacrificed at ZT6. ID numbers of sections: *per1 *control: 145-0202054795 at 3625; SD: 153-0202055093 at 3825; *per2 *control: 140-0205056163 at 3500; SD: 132-0205058042 at 3300. Lower right panel is the histological view (Nissl stain) of the upper right panel (*per1-*SD) with cerebral cortex (CTX), thalamus (TH), hypothalamus (HY), and cerebellum (CB). The presence of EEG electrodes in some mice caused cortical damage during brain removal.

To gain insight into which forebrain regions contributed to the SD-induced changes in *per1 *mRNA levels reported above, we performed *in situ *hybridizations. During control conditions, forebrain expression levels were low, especially in the cerebral cortex, while expression in the cerebellum was high (Fig.4), matching previously published studies [12,13,16,34]. Following 6 h SD, *per1 *expression increased throughout the brain (Fig.4). This increase was especially pronounced in the cerebral cortex, consistent with our qPCR data (Fig.1). Within the cortex, increases were especially notable in the frontal cortex and in and around the cingulate cortex. Expression in the cerebellum was also importantly increased. Expression patterns and effects of SD described here for *per1 *match those of *per1 *and *per2 *publicly available from the Allen Institute for Brain Science website [35] (Fig.5).

## Discussion

We previously found that cortical mRNA levels of the clock genes *per1 *and *per2 *were increased after SD in rats and B6 mice [4]. Here we have extended that observation in two additional inbred strains; AK and D2. Furthermore, by following the time course of these changes during SD and recovery sleep, we established that *per *expression changes as a function of the time-spent-awake and asleep. Such a time course is consistent with a role for these genes in the homeostatic regulation of sleep since the need for sleep increases with duration of wake and decreases with duration of sleep. A novel aspect of this study is the comparison of the SD-induced changes in gene expression among three inbred strains for which the homeostatic regulation of sleep differs [25]. During both spontaneous and enforced periods of wakefulness the need for sleep, indexed by the level of EEG delta power, increased fastest in AK, intermediate in B6, and slowest in D2 mice. If indeed *per *expression would simply parallel EEG delta power we would expect to find the largest increase in *per *expression after SD in AK mice and the smallest in D2. Here we found evidence for the opposite; although *per *expression was consistently increased with SD, this increase was modulated by genotype and was found to be highest in D2 and lowest in AK. In addition, *per2 *expression remained elevated during recovery sleep in D2 mice only. Thus high, sustained levels of *per2 *expression might negatively impact the sleep recovery process and/or expression of EEG delta oscillations during NREMS.

An implicit assumption in our interpretation of our results is that the sleep-wake dependent changes in the expression of *per1*, *per2*, and *dbp *are mediated by CLOCK and/or NPAS2 induced transcription. The observation in *npas2*^-/- ^mice that cortical *per2 *expression no longer follows the circadian sleep-wake distribution [12], together with the observations that the increase in *per2 *expression after SD is reduced in these mice [3], and that the homeostatic response to SD is altered in *npas2*^-/-^, *cry1,2*^-/-^, and *bmal1*^-/- ^mice (see Introduction) strongly support this assumption. Nevertheless, expression of the genes we examined can be induced by other transcriptional regulators as well. E.g., *per1 *can be readily induced by cAMP-responsive element-binding protein (CREB) [29,36,37]. CREB signaling also increases during prolonged wakefulness [38,39] and thus might have contributed to the SD-dependent changes in *per1 *reported here. Another example is the close link between glucocorticoid signaling and *per1 *and *per2 *expression [40-43] through which SD, which is accompanied by an increase in plasma corticosterone levels, could have affected *per *expression. In fact, we found that the SD-induced increase in corticosterone levels was 3-fold higher in D2 mice compared to the other two strains (Franken *et al*., in preparation) consistent with the larger relative increase in *per *expression in this strain observed here. Evidence for an altered transcriptional regulation of the four genes during SD (compared to that during control conditions) is provided by the altered relationship between the expression patterns we report here.

The cortical expression of *dbp *was decreased after SD confirming previously published data [4,44]. Given the fact that *per1*, *per2*, and *dbp *transcription are, in part, under the same transcriptional control, these opposite effects of the SD are not easily explained. PER proteins are co-repressors of CLOCK and NPAS2 induced transcription. Thus, assuming that increased *per *expression is followed by increased PER levels within the time frame of the SD, PER-mediated transcriptional repression is an obvious possible pathway by which *dbp *expression could be decreased. In line with this assertion, we found that the decrease in *dbp *expression to be largest in the strain with the largest increase in *per *expression; i.e. D2. The observation that *per *and *dbp *expression were nevertheless positively correlated during the SD is, however, inconsistent with this scenario. In addition, *per *expression itself could be expected to decrease as well, at least the contribution of CLOCK and NPAS2 mediated transcription to the total *per *mRNA. Finally, the time course of the peripheral changes in *dbp *and *per *expression under control conditions also doesn't support a *dbp *suppressing role for PER. *dbp *mRNA levels peak at ZT8 and are declining when *per *mRNA starts to increase [16,26-28].

The *in situ *hybridization studies indicated that SD induced pronounced changes in *per *expression in cerebellum and cerebral cortex. This is in line with our previous findings using qPCR showing that *per *expression was increased in the cerebral cortex but not in brainstem and hypothalamus [4] and with the *per2 *increase observed after SD both in cerebellum and cerebral cortex [44]. While the *in situ *data are limited, we observed some of the highest expression and largest increases in *per1 *mRNA in the most frontal area of the cortex. EEG delta power is generally higher and its increase after SD more pronounced in frontal cortex relative to other cortical areas such as occipital regions [45-47] in accordance with the concept that frontal areas in particular are affected by SD [48]. In mice lacking a functional PER1 protein (*per1*^Δ/Δ^) EEG delta power was decreased in the frontal derivation specifically and the SD-induced increase in EEG delta power was smaller in *per1*^Δ/Δ ^and *per2*^Δ/Δ ^mice also only in the frontal derivation [49].

Already clear from the present data is that not all changes in forebrain *per *expression can be attributed to changes in the sleep-wake distribution; expression increases during the latter part of the light period while animals remain mostly asleep. This increase precedes dark onset after which maximal *per *expression levels are reached when mice are the most active [16,27]. A major adaptive advantage provided by circadian rhythms is that it allows organisms to anticipate and prepare for predictable periodic changes in the environment [50]. The anticipatory increase in *per *expression might be part of a molecular repertoire preparing the animal for the active period. Some types of anticipatory behavior, however, don't require an intact circadian system, as food-restriction studies have shown [51]. Moreover, clock-genes in the forebrain seem to play a permissive role in such anticipatory behaviors. For example, *npas2*^-/- ^mice, which have intact circadian rhythms in overt behavior, have difficulty adapting to and anticipating altered feeding schedules [17]. Besides food intake, other homeostatically regulated behaviors such as sleep seem to be similarly affected [3]. Thus, the same molecular network of transcriptional regulators that sets internal time-of-day in the SCN might, in the forebrain, track and signal time without sleep (or food). Conceptually the distinction between homeostatic and circadian processes at the molecular level thus becomes unclear. Mechanistically we proposed that changes in clock-gene expression could be tied directly to cellular energy state based mainly on the observation that CLOCK and NPAS2 mediated transcription is sensitive to redox state [22,23]. In this respect the lack of an effect of SD on *ldha *expression is disappointing. Although *ldha *has a canonical E-box and has been shown to be activated by both NPAS2 and CLOCK in a neuroblastoma cell line [22], mRNA levels of this gene were surprisingly stable across time-of-day, experimental condition, and genetic background.

## Conclusion

We found that the expression of three circadian clock genes changes as a function of time-spent-awake and that these changes were influenced by strain. These results further indicate that circadian clock genes play a role in the homeostatic regulation of sleep. Additional evidence in support of our hypothesis has recently been obtained in humans homozygous for one of two known polymorphisms in *hPer3 *[52]. Based on SCN lesion experiments in rats [53] and forced desynchrony studies in humans [54] it is generally thought that homeostatic and circadian processes operate separately and have different neurophysiological substrates. Our current results underscore that, at least at a molecular level, these two processes are difficult to separate. Recent data also show that at the neuro-physiological level, SD can affect the activity of SCN neurons previously thought to be dedicated exclusively to setting internal time-of-day[55]. Finally, the present results might be of relevance for research on the biology of mood disorders. In this field there is increasing interest in the relation between clock-genes and e.g. seasonal affective disorders [56] or bipolar disorders [57]. The effects of sleep deprivation on clock-gene expression demonstrated here combined with the fact that sleep deprivation is a well known antidepressant add a molecular component to the intricate relationship between mood, circadian rhythms, and sleep (reviewed in [58,59]).

## Methods

### Animals and Experimental Protocol

A total of 102 mice contributed to this study. Of those, 96 were used for quantitative PCR (qPCR) analyses of *per1*, *per2*, *dbp*, and *ldha *expression in AKR/J (AK), C57BL/6J (B6), or DBA2/J (D2) mice (n = 32/genotype). The remaining 6 B6 mice were used for in situ hybridization studies of *per1*. All mice were 11-week old males which were kept under a 12 h light/dark cycle for at least 14 days before the experiments. For the qPCR experiment half of the mice (the SD-group) were sleep deprived by gentle handling during the light phase which is their usual rest phase. The other half served as controls and were left undisturbed so they could sleep *ad lib*. SD started at light-onset (Zeitgeber time, ZT0). Mice of the SD-group were sacrificed after 1-, 3-, or 6 h SD (i.e., at ZT1, -3, or -6). A 4th group was allowed 2 h of recovery sleep after 6 h SD (ZT8). At each time point 4 mice per genotype were taken. An equal number of control group mice were sacrificed at these 4 time points (n = 4/time-point/group/genotype). For *in situ *hybridization studies 3 mice were sleep deprived for 6 h starting at ZT0; the remaining 3 served as controls. Animals of both groups were sacrificed at ZT6.

### qPCR

Brains were rapidly removed, dissected, and frozen at -70°C. Dissection involved removal of the cerebellum and pons-medulla, followed by a single mid-sagittal cut through the remaining brain (referred to as forebrain) producing a left and a right hemisphere. Total RNA from mouse forebrains was extracted and purified using TRIzol^® ^Reagent(Invitrogen). RNA pellets were resuspended and incubated in RNAsecure™ (Ambion). Prior to cDNA synthesis, RNA samples were treated with Turbo DNA-*free*™ (Ambion) to remove genomic DNA contamination, and three micrograms of total RNA were converted into first strand cDNA with Oligo(dT)_12–18 _and Superscript II™ (Invitrogen).

To determine gene expression profiles, cDNA of individual mice was diluted 1:4 and 2 μl was aliquots were amplified in a 50 μl reaction mixes containing 25 μl of iQ™ SYBR^® ^Green Supermix (BioRad) and 0.2 μM each of forward and reverse gene-specific primers. Samples were placed into 96-well 0.2 thin-wall PCR plates (BioRad), covered with optical-quality sealing tape (Biorad), and subjected to 90s at 95°C and then subjected to 40 cycles of 15s at 94°C, 60s at 60°C, and 1 min at 72°C in an iCycler iQ™ real-time Detection System (BioRad). After PCR, products were subjected to a melt curve protocol to ensure that primer-dimers and non-specific products are not present. Briefly, PCR reaction products were melted at 95°C for 15s, then the temperature was lowered to 60°C for 1 min and then the temperature increased in increments at 0.5°C per 10s over 80 cycles. SYBR binds to the double-stranded PCR product until the Tm of the product is reached. Then, a sharp decline in fluorescence occurs resulting in a single peak as long as only one PCR product was amplified (gel electrophoresis of PCR products also confirmed a single band for all primer sets). The following forward and reverse primers used in this experiment were designed with Primer3, synthesized (Sigma-Genosys), and utilized as described above: *per1 *forward 5'-CTTGATGTGATGGCGTGTGT-3', reverse 5'-AGCTGGGGCAGTTTCCTATT-3', *per2 *forward 5'-ATCTCCAGGCGGTGTTGAAG-3', reverse 5'-AGGGTTACGTCTGGGCCTCT-3', *dbp *forward 5'-AAGGCAAGGAAAGTCCAGGT-3', reverse 5'-TGGGACAAGGAGGAAGTCAG-3', *ldha *forward 5'-GTGCCTGTGTGGAGTGGTGT-3', reverse 5'-TGCAGCCTGGACAGTGAAGT-3', *gapdh *forward 5'-CCATCAACGACCCCTTCATT-3', reverse 5'-TCTCGTGGTTCACACCCATC-3', *actb *forward: 5'-GCCGGGACCTGACAGACTAC-3', reverse 5'-ATGGTGCTAGGAGCCAGAGC-3'.

Relative abundance of each gene was normalized to both *gapdh *and *actb *determined by the relative standard curve method in which undiluted cDNA of all samples in each experiment were pooled and serial dilutions of the pool (stock, 1:5, 1:10, 1:100, and 1:1000) were run simultaneously with the unknowns in each assay. Each unknown sample was diluted 1:4 and run in duplicate and mean expression relative to the common pool for each experiment was determined.

### per1 in situ hybridization

Brains were rapidly removed, frozen on dry ice, and stored at -70°C until they were coated in embedding medium (Optimal Cutting Temperature Compound, Tissue Tek 4583) and sectionedhorizontally on a cryostat at 15 μm. Sections from control and SD mouse brainswere thaw mounted on micro-slides (pre-cleaned Superfrost Plus, VWR Scientific), fixed in 4% paraformaldehyde and dehydrated through an ethanol series and stored at -70°C.

Anti-sense *per1*RNA probes were made froma linearized mouse cDNA clone in pBluescript (KS^-^) (Stratagene, La Jolla, CA). The ^35^S-labeled *per1 *probe was transcribed from template DNA with T7 RNA polymerasein a reaction containing 5 mM each of CTP, ATP, and GTP; 10 mM dithiothreitol (DTT); 1X Transcription Buffer (Boehringer Mannheim, Indianapolis, IN); RNasin (Promega, Madison, WI); ^35^S-UTP (> 1,000 CI/mmol; NEN, Boston, MA). Probes were incubated with hydrolysis solution (50 mM DTT, 40 mM NaHCO_3_, 60 mM Na_2_CO_3_) for 1 h at 37°C, followed by treatment with neutralization solution (50 mM DTT, 5% acetic acid, 100 mM sodium acetate). Probes were then precipitated and stored at -70°C.

Sections were thawed to room temperature, rehydrated, incubated with 20 g/ml proteinase K, and treated with acetic anhydride in 0.1 M triethanolamine. Dehydrated sections were hybridized for 12 h at 60°C with 5 × 10^6 ^cpm/ml ^35^S-labeled *per1 *RNA anti-sense probe in hybridization buffer [50% formamide, 300 mM NaCl, 20 mM Tris HCL (pH 7.4), 5 mM EDTA, 10 mM NaPO_4_(pH 8.0), 100 mM DTT, 10% dextran sulfate 1 × Denhardt, 50 g/ml total yeast RNA] under siliconized coverslips. Coverslips were floated off in 4 × SSC at room temperature, followed by four washes in 2 × SSC, DTT. Slides were rinsed in washing buffer [400 mM NaCl, 10 mM Tris HCL (pH 7.5), 5 mM EDTA] and incubated with 20 g/ml RNAse A in washing buffer at 37°C, followed by washes in 2 × SSC and 0.1 × SSC. Slides were dehydrated and placed on autoradiography film (Kodak Hyperfilm MP) for direct imaging to reveal areas of RNA expression resulting from annealed ^35^S-labeled anti-sense probes. Film was developed after 18-day autoradiography film exposure.

## Authors' contributions

PF initiated and designed experiments, analyzed data, and wrote manuscript. RT conducted experiments, developed methods, and analyzed data. HCH was involved in experimental design, lab direction, and editing manuscript. BFO, along with PF, initiated and designed experiments, analyzed data, directed all experimental work by RT, and co-wrote manuscript. All authors have read and approved the final article.
